# Low colorectal cancer survival in the Mountain West state of Nevada: A population-based analysis

**DOI:** 10.1371/journal.pone.0221337

**Published:** 2019-08-19

**Authors:** Karen E. Callahan, Carmen P. Ponce, Chad L. Cross, Francisco S. Sy, Paulo S. Pinheiro

**Affiliations:** 1 Department of Environmental and Occupational Health, School of Public Health, University of Nevada Las Vegas, Las Vegas, Nevada, United States of America; 2 Nevada Central Cancer Registry, Nevada Division of Public and Behavioral Health, Carson City, Nevada, United States of America; 3 Department of Radiation Oncology, School of Medicine, University of Nevada Las Vegas, Las Vegas, Nevada, United States of America; 4 Sylvester Comprehensive Cancer Center, Miller School of Medicine, University of Miami, Miami, Florida, United States of America; 5 Department of Public Health Sciences, Miller School of Medicine, University of Miami, Miami, Florida, United States of America; Leibniz Institute for Prevention Research and Epidemiology BIPS, GERMANY

## Abstract

Colorectal cancer (CRC) is the third greatest cancer burden in the United States. The remarkably diverse Mountain West state of Nevada has uncharacteristically high CRC mortality compared to other Western states. We aimed to study the determinants of the CRC excess burden by using data from the Nevada Central Cancer Registry from 2003–2013. Five-year cause-specific age-adjusted survival from colorectal cancer was calculated and stratified by sex, race/ethnicity and region of Nevada. Cox Proportional Hazards regression modelling was used to study the impact of demographic, social, and clinical factors on CRC survival in Nevada, assessing follow-up as accurately as possible. The extent to which differences in survival can be explained by receipt of stage-appropriate treatment was also assessed. 12,413 CRC cases from 2003–2013 in Nevada were analyzed. Five-year CRC survival was low: 56.0% (95% CI: 54.6–57.5) among males and 59.5% (95% CI: 58.0–61.1) among females; significantly lower than national 5-year survival of 65.1% and 66.5%, respectively. Low survival was driven by populous Southern Nevada; after adjustment for all covariates, Southern Nevadans were at 17% higher risk of death than their counterparts in Northwestern Nevada (HR:1.17; 95% CI:1.08–1.27). Many patients did not receive stage-appropriate treatment, although this only partly explained the poor survival, uniformly low for every race/ethnicity in Nevada. The observed disparity for this one state within a single nation merits public health attention; regardless of the state or region of residence, all Americans deserve equal opportunity for optimum health outcomes in the face of a cancer diagnosis. The current study provides baseline information critical to clinicians, public health professionals, and all relevant stakeholders as they attempt to discern why Nevada’s outcomes are vastly divergent from its neighboring Western states and make plans for remediation.

## Introduction

Colorectal cancer (CRC) is the third leading cause of cancer incidence and mortality among both men and women in the United States (US) [1], with an estimated 145,600 new cases and 51,000 deaths expected in 2019 [1]. It currently accounts for between 8–9% of all US cancer cases and deaths [2]. While rate declines in mortality have been seen in recent decades, attributable both to improvements in treatment and to the increased reach of CRC screening on a population basis [3], the CRC burden across the US—as measured by incidence, survival, and mortality—is not distributed uniformly. Disparities are seen by race/ethnicity, sex, socioeconomic status, and geography. Non-Hispanic black and American Indian/Alaskan Native populations suffer disproportionately high CRC incidence and mortality compared to non-Hispanic whites; in aggregate, Hispanics and Asian/Pacific Islanders (API) fare better [3]. Rural populations as well as those with low socioeconomic status shoulder a higher burden [4–6] and have benefitted least from advances in screening and treatment [7, 8]. Broad trends across the US suggest that the highest CRC burden is seen in the Southeast and the lowest is seen in the Western region of the United States [9].

The Mountain West state of Nevada is a unique state, the 7^th^ largest by land area, comprised of only two large metropolitan areas and vast swaths of rural and frontier lands. The current population of almost 3 million reflects tremendous population growth, over 50% since 2000 [10], contributing to Nevada’s strikingly diverse profile. Approximately half of Nevada’s population identifies as minority, including 10% non-Hispanic black, 9% Asian/Pacific Islander, and 30% Hispanic [10]. Moreover, 20% of Nevada’s population is foreign-born, ranking fifth of all states [10]; it also leads all other states in the proportion of its total population that is undocumented: approximately 7% [11]. In addition to the unique geographic and demographic profile, Nevada consistently ranks low on two important social indicators of health: education [12] and health insurance coverage [13].

According to known cancer patterns, Nevada stands as an exception, with an uncharacteristically high CRC mortality rate compared to any Western state, including its five neighbors: Arizona, California, Idaho, Oregon, and Utah [3]. However, to date, only one outdates study attempted to characterize CRC survival in Nevada [14].

Therefore, this study aims to accurately characterize colorectal cancer survival in Nevada by race/ethnicity, sex, stage at diagnosis, and region of Nevada, and compare with national survival. We also aim to identify within-state survival disparities and examine the impact of available demographic, social, and clinical factors on CRC survival. Owing to its rapidly increasing population, understanding what drives Nevada’s unfavorable CRC profile is critical to meeting the cancer control and prevention needs of Nevada’s burgeoning and racial/ethnically diverse population.

## Methods

The Institutional Review Board at the University of Nevada, Las Vegas reviewed this study, #858093–1, and determined it was exempt. All data were de-identified.

All cases of first primary colorectal cancer, inclusive of International Classification of Diseases for Oncology (ICD-O-3) codes C18-C20 [15], diagnosed in the state of Nevada between 2003–2013 (all years available at time of study) were included in this project; excluded cases were those diagnosed by death certificate or autopsy only (n = 306), as well as cases with a negative or missing survival period (n = 17). Data were obtained from the Nevada Central Cancer Registry (NCCR), which collects all cancer incidence data in Nevada using standards established by the Centers for Disease Control and Prevention’s National Program of Cancer Registries [16] and the North American Association of Central Cancer Registries [17]. To minimize the number of missing deaths, customary death record linkages with the Nevada Office of Vital Records were augmented by linkage with the National Death Index [18]. For comparison with national survival, CRC survival for the same time period was derived from the National Cancer Institute’s Surveillance, Epidemiology, and End Results Program (SEER) data. SEER-18 covers approximately 28% of the US population, and is fairly representative of the entire US cancer population; it does not contain Nevada data [19].

Covariates included demographic, social, and clinical factors ascertained at date of diagnosis and assessed for their impact on colorectal cancer survival. Age was categorized into the following five groups: 15–44, 45–54, 55–64, 65–74, and 75+, according to the International Cancer Survival Standards age classification [20]. Cases were classified into six mutually exclusive racial/ethnic groups: non-Hispanic white, non-Hispanic black, Hispanic, American Indian/Alaska Native (AI/AN), Filipino, and other API (Chinese, Japanese, Korean, Indian, etc.). Filipinos were considered separately from other APIs as they accounted for 67% of Nevada’s overall API population in 2010 [21]. Place of residence in Nevada was divided into three distinct geographic regions. Southern Nevada is comprised of populous Clark County, which includes Las Vegas and the surrounding areas. The Northwestern Nevada region represents the only other large population area in NV, encompassing the capital, Carson City, as well as Douglas, Lyon, Storey, and Washoe counties. The remaining eleven sparsely populated rural and frontier counties were classified as Rural Nevada.

Social factors included were insurance type, marital status, and socioeconomic status (SES). Insurance categories included private, Medicare, Medicaid, uninsured, and unknown. Marital status categories were married, single, divorced/separated, widowed, or unknown. Since SES information is not directly collected on individual patients by the NCCR, the proportion of people living in poverty in 2011 in a patient’s zip code of residence at diagnosis was used as a proxy for SES; less than 5% poverty in zip code of residence was considered high SES, between 5–10% was medium SES, and over 10% was low SES.

Tumor stage at diagnosis was coded I, II, III, or IV, according to guidelines set by the American Joint Committee on Cancer [22]. However, for comparison with national survival estimates, SEER Summary Staging of localized, regional, and distant groups was used [23]. Anatomic sublocations considered were right colon (C18.0–18.3), left colon (C18.4–18.7), colon NOS (C18.8, C18.9) and rectum (C19.9, 20.9). Morphologies included adenocarcinomas (ADKs), ADKs in adenoma/polyp, Mucinous ADKs, Carcinoids, and Carcinoma NOS.

Receipt of stage-appropriate treatment was determined only for AJCC Stage I-III tumors by assessing whether treatments (radiation, chemotherapy and/or surgery) were received in accordance with guidelines developed by The American Society of Colon and Rectal Surgeons [24, 25], as presented in Table 1. Cases with missing information for any treatment variable required for determination of stage-appropriate treatment were considered Unknown. Stage IV tumors were excluded for this sub-analysis since registry data does not distinguish whether treatments are used for palliative or curative intent.

**Table 1:** Minimum requirements for consideration as having received stage-appropriate treatment^a^ for AJCC Stages I-III.

Stage at Diagnosis	Colon Cancer	Rectal Cancer
	C18 (all)	C19.9 & C20.9
AJCC I	surgery	Surgery
AJCC II	surgery	surgery, radiotherapy & chemotherapy
AJCC III	surgery & chemotherapy	surgery, radiotherapy & chemotherapy

Abbreviation: AJCC, American Joint Committee on Cancer.

a. Guidelines developed by The American Society of Colon and Rectal Surgeons.

Five-year cause-specific age-adjusted survival from colorectal cancer was calculated and stratified by sex, race/ethnicity (all-stages-combined), and region of Nevada (all-stages-combined and stage-specific), using the life table method. Survival time was measured in months from the date of diagnosis until either the date of death or the end of the study period, Dec 31, 2014, whichever occurred first. The presumed alive assumption [26] was used for censored observations; cases dead from another cause prior to the end of the study were censored. Survival from SEER-18 data was computed using the presumed alive option for comparability with Nevada survival.

Kaplan-Meier survival curves were developed to visualize differences in survival within the state of Nevada by stage at diagnosis (SEER summary staging), period of diagnosis (2003–2007 and 2008–2012), race/ethnicity, and region of Nevada. Log-rank tests were used to detect differences in survival curves.

To calculate comparative risk of death and identify factors impacting survival, Cox proportional hazards regression models for multivariate survival analysis were constructed, adjusting for all covariates significant in univariate analyses. Hazard ratios and their corresponding 95% confidence intervals (CI) were computed. Separate models were constructed for the sub-analysis to examine risk of CRC death for cases diagnosed in AJCC stages I-III (with complete information on treatments) by region of Nevada and receipt of stage-appropriate treatment.

Statistical tests were interpreted as significant against α = 0.05. Data were analyzed with SPSS v22 and SAS 9.3.

## Results

In the 11-year period of 2003–2013, first primary colorectal cancer tumors were identified in 12,413 patients from the Nevada Central Cancer Registry. Fifty-five percent of patients were male, 61% were over 65 years of age, 78% were non-Hispanic white and two-thirds were from Southern Nevada. Almost half were married; 73% had either private insurance or Medicare. Over 90% of tumors were some type of ADK; distribution between right colon, left colon, and rectum was approximately equal but almost 10% were colon NOS cases. For the sub-analysis, 68% of tumors (n = 8480) were diagnosed in AJCC Stages I, II, or III; of those, only 36% received stage-appropriate treatment, while 39% did not. For 26% of these cases, stage-appropriate treatment was unable to be determined due to missing treatment information (Table 2).

**Table 2:** Characteristics of 12,413 colorectal cancer cases from 2003–2013 in Nevada.

DEMOGRAPHIC FACTORS		N	%
**Sex**	Male	6817	54.9
	Female	5596	45.1
**Age Group**	16–44 years old	583	4.7
	45–54 years old	1519	12.2
	55–64 years old	2730	22.0
	65–74 years old	3589	28.9
	75+ years	3992	32.2
**Race/Ethnicity**	Non-Hispanic white	9674	77.9
	Non-Hispanic black	927	7.5
	Hispanic	1041	8.4
	Filipino	220	1.8
	Other Asian/Pacific Islander	467	3.8
	American Indian/Alaskan Native	84	0.7
**Nevada Region**	Northwestern Nevada	2714	21.9
	Southern Nevada	8223	66.2
	Rural Nevada	1025	8.3
	Unknown	451	3.6
**SOCIAL FACTORS**			
**Marital Status**	Married	6008	48.4
	Single	1887	15.2
	Divorced/Separated	1284	10.3
	Widowed	1899	15.3
	Unknown	1335	10.8
**Insurance Status**	Private Insurance	4470	36.0
	Medicare	4579	36.9
	Medicaid	602	4.8
	Uninsured	470	3.8
	Unknown	2292	18.5
**Socioeconomic Status**	High	2453	19.8
	Intermediate	4222	34.0
	Low	4878	39.3
	Unknown	860	6.9
**CLINICAL FACTORS**			
**Stage at Diagnosis**	AJCC I	2429	19.6
	AJCC II	2798	22.5
	AJCC III	3253	26.2
	AJCC IV	2097	16.9
	Unknown	1836	14.8
**Morphology**	Adenocarcinoma (ADK)	9614	77.5
	ADK in adenoma/polyp	481	3.9
	Mucinous ADK	1173	9.4
	Carcinoids	307	2.5
	Carcinoma NOS	838	6.8
**Sublocation of Tumor**	Colon-Right	3902	31.4
	Colon-Left	3767	30.3
	Rectum	3588	28.9
	Colon NOS	1156	9.3
**STAGE-APPROPRIATE TREATMENT**^**a**^		**n = 8480**	**%**
**Received**	Yes	3028	35.7
	No	3263	38.5
	Unknown^b^	2189	25.8

Abbreviations: AJCC, American Joint Committee on Cancer; ADK, Adenocarcinoma; NOS, Not Otherwise Specified.

a. Restricted to AJCC Stages I,II,III;

b. Unable to make determination.

**Fig 1:**
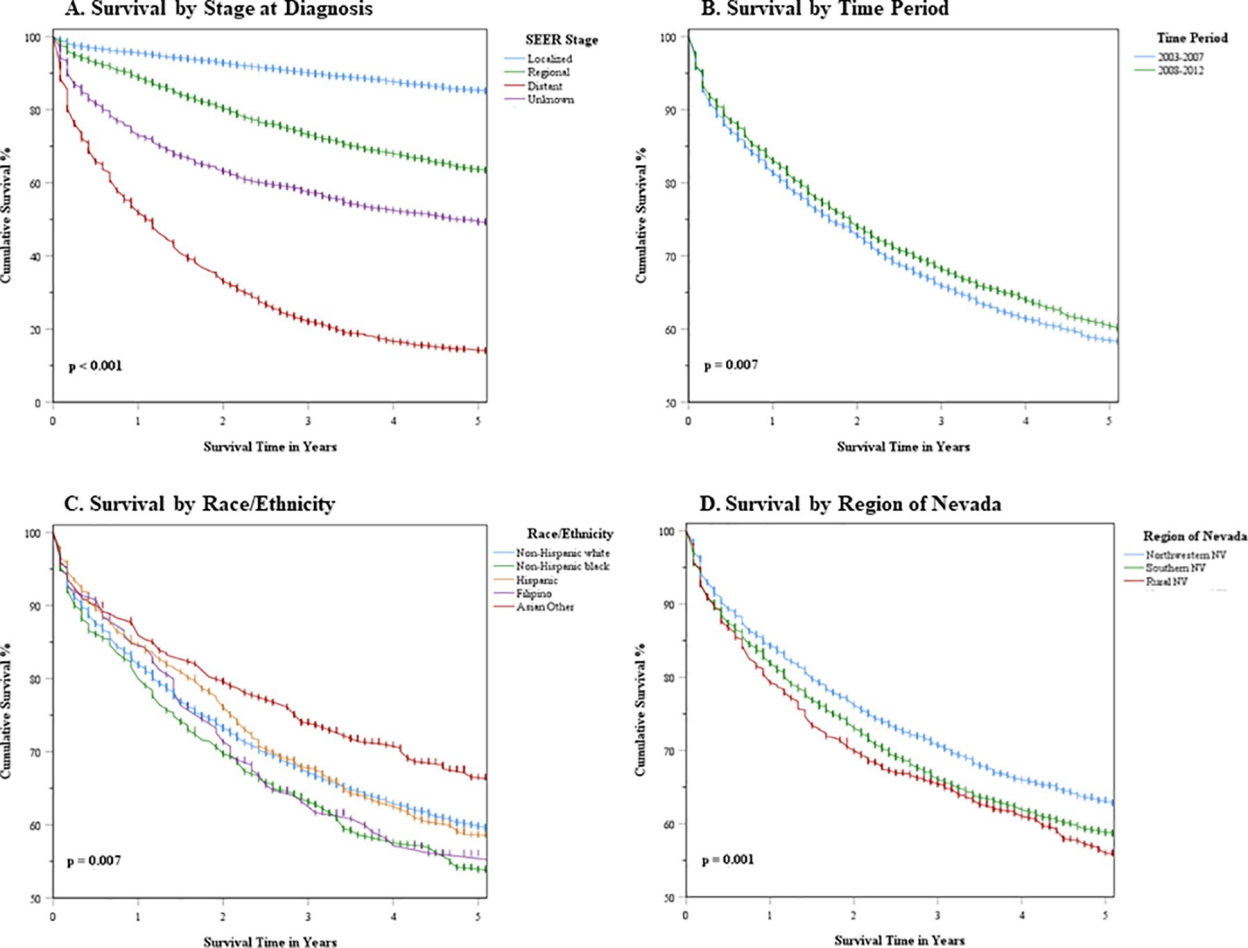
Kaplan Meier CRC survival curves for select prognostic factors in Nevada. Survival differences by: A. stage at diagnosis B. period of diagnosis C. race/ethnicity D. region of Nevada.

As expected, Kaplan Meier curves showed significantly better survival from CRC in Nevada for tumors diagnosed in localized and/or regional stages (p < .001). Moreover, survival was slightly higher in the more recent 2008–2012 time period than in 2003–2007 (p = .007). Significant survival differences were also seen for race/ethnicity and region of Nevada (Fig 1).

Five-year age-adjusted colorectal-cancer-specific survival in Nevada was 56.0% (95% CI: 54.6–57.5) among males, significantly lower than females at 59.5% (95% CI: 58.0–61.1); both were significantly lower than corresponding national proportions of 65.1% and 66.5% based on the SEER-18 catchment area for the same time period. Within Nevada, non-Hispanic black males had the lowest survival of any analyzed group, at 48.3% (95% CI: 42.7–53.9), and Other API females had the highest at 68.8% (95% CI: 61.6–76.0). However, for every distinct male racial/ethnic group, CRC survival in Nevada was significantly lower than CRC survival of their counterparts nationally. Likewise, non-Hispanic white and Hispanic females had significantly lower survival compared to SEER-18 CRC patients of the same race/ethnicity (Table 3).

**Table 3:** Five-year age-adjusted^a^ colorectal cancer survival by sex and by race/ethnicity^b^. Nevada compared to overall United States^c^. 2003–2013.

		Male		Female	
		Survival	95% CI	Survival	95% CI
**All race/ethnicities**	Overall US	65.1%	(64.8–65.3)	66.5%	(66.3–66.8)
	Nevada	56.0%	(54.6–57.5)	59.5%	(58.0–61.1)
**Non-Hispanic white**	Overall US	65.9%	(65.6–66.2)	67.2%	(66.9–67.5)
	Nevada	59.3%	(57.7–61.0)	59.9%	(58.1–61.6)
**Non-Hispanic black**	Overall US	56.0%	(55.0–56.9)	60.3%	(59.5–61.1)
	Nevada	48.3%	(42.7–53.9)	54.3%	(48.6–60.1)
**Hispanic**	Overall US	64.3%	(63.4–65.1)	66.6%	(65.6–67.4)
	Nevada	57.0%	(51.9–62.1)	56.0%	(50.7–61.4)
**Filipino**	Overall US	67.7%	(65.5–69.7)	69.6%	(67.4–71.6)
	Nevada	46.1%	(34.2–58.0)	63.2%	(52.9–73.5)
**Other API**	Overall US	69.3%	(68.2–70.3)	70.0%	(68.9–71.0)
	Nevada	59.8%	(52.1–67.6)	68.8%	(61.6–76.0)

Abbreviations: API, Asian/Pacific Islander; CI, Confidence Interval; US, United States.

a. Adjusted according to International Cancer Survival Standard.

b. American Indian/Alaskan Native suppressed due to small numbers

c. SEER-18.

Examining by stage at diagnosis, five-year CRC survival in the entire state of Nevada for localized and regional stage tumors was significantly lower than national survival. Males and females in Nevada diagnosed with CRC at a localized stage had 5-year survival proportions of 83.3% and 86.7%, respectively, compared to national survival of 88.6% (males) and 90.0% (females). Even larger survival differences were seen for males and females in Nevada diagnosed with CRC at a regional stage; 5-year survival was 61.9% and 63.2%, respectively, compared to national survival of 69.9% (males) and 71.4% (females). Survival for distant stage CRC tumors was not significantly different than national survival for either sex. Notably, by geographic region of Nevada, stage-specific survival in Northwestern Nevada did not differ from national survival at any stage for both men and women. Five-year CRC survival in Southern Nevada was significantly lower than national survival for all diagnosis stages except for distant stage in men (Table 4).

**Table 4:** Five-year age-adjusted^a^ all stage & stage-specific colorectal cancer survival by geographic region of Nevada compared to overall United States^b^. 2003–2013.

	All Stages		Localized Stage		Regional Stage		Distant Stage	
MALE	Survival	95% CI	Survival	95% CI	Survival	95% CI	Survival	95% CI
**Overall United States**^**b**^	65.1%	(64.8–65.3)	88.6%	(88.3–88.8)	69.9%	(69.4–70.4)	13.0%	(12.5–13.4)
**Nevada**	56.0%	(54.6–57.5)	83.3%	(81.3–85.3)	61.9%	(59.6–64.2)	11.8%	(9.5–14.0)
Northwestern Nevada	58.8%	(55.7–61.9)	85.2%	(81.2–89.3)	64.8%	(60.0–69.6)	15.2%	(10.1–20.2)
Southern Nevada	55.8%	(54.0–57.5)	82.3%	(79.8–84.8)	61.8%	(58.9–64.6)	10.2%	(7.5–12.9)
Rural Nevada^c^	52.2%	(47.0–57.4)	-	-	-	-	-	-
**FEMALE**								
**Overall United States**^**b**^	66.5%	(66.3–66.8)	90.0%	(89.7–90.3)	71.4%	(71.0–71.8)	15.2%	(14.7–15.7)
**Nevada**	59.5%	(58.0–61.1)	86.7%	(84.5–88.8)	63.2%	(60.8–65.7)	14.0%	(11.3–16.7)
Northwestern Nevada	63.8%	(60.6–67.0)	89.1%	(85.1–93.1)	66.4%	(61.3–71.6)	21.3%	(15.0–27.7)
Southern Nevada	58.3%	(56.4–60.2)	85.9%	(83.3–88.6)	62.0%	(59.0–65.0)	10.5%	(7.4–13.5)
Rural Nevada^c^	58.4%	(52.6–64.3)	-	-	-	-	-	-

a. Adjusted according to International Cancer Survival Standard.

b. SEER-18.

c. Rural data were too sparse to accurately calculate age-adjusted survival by stage.

After adjustment for all clinical, social, and demographic covariates, as well as year of diagnosis, Cox proportional hazard model estimates indicated that female CRC patients were at 11% lower risk of death than male. Southern Nevadans were at 17% higher risk than their counterparts in Northwestern Nevada (HR:1.17; 95% CI:1.08–1.27). By race/ethnicity, non-Hispanic blacks and Filipinos did not differ significantly from non-Hispanic whites, while Hispanics and Other APIs had 14% and 25% significantly lower risk, respectively (HR: 0.86; 95% CI: 0.77–0.97) and (HR: 0.75; 95% CI: 0.63–0.90). Patients with any insurance status type other than private insurance had higher risk of CRC death. Likewise, any marital status besides the referent, married, conferred significantly higher risk of death. CRC mortality risk increased inversely with socioeconomic status. For clinical factors, carcinoma NOS cases had 74% higher risk than the referent ADK morphology. By tumor sublocation, patients with colon NOS cases had an almost 50% higher risk of death than those with right-sided tumors, while risk of death from a left-sided CRC tumor was lower than from a right-sided tumor (HR: 0.89; 95% CI: 0.82–0.97). Patients with metastatic CRC, AJCC stage IV, had a 14 times higher risk of death than those with Stage I (Table 5).

**Table 5:** Demographic, social, and clinical determinants of CRC risk of death^a^ in Nevada, 2003–2013.

		HR	95% CI
**Sex**	Male	*referent*	
	Female	.89	(0.83–0.95)
**Nevada Region**	Northwestern Nevada	*referent*	
	Southern Nevada	1.17	(1.08–1.27)
	Rural Nevada	1.11	(0.98–1.27)
**Race/ Ethnicity**^**b**^	Non-Hispanic white	*referent*	
	Non-Hispanic black	0.96	(0.86–1.09)
	Hispanic	0.86	(0.77–0.97)
	Filipino	0.92	(0.74–1.16)
	Other Asian/Pacific Islander	0.75	(0.63–0.90)
**Marital Status**	Married	*referent*	
	Single	1.14	(1.04–1.25)
	Divorced/Separated	1.26	(1.14–1.40)
	Widowed	1.27	(1.15–1.40)
**Insurance Status**	Private Insurance	*referent*	
	Medicare	1.16	(1.07–1.27)
	Medicaid	1.38	(1.20–1.59)
	Uninsured	1.43	(1.24–1.66)
**Socioeconomic Status**	High	*referent*	
	Intermediate	1.22	(1.11–1.34)
	Low	1.38	(1.26–1.52)
**Stage at Diagnosis**	AJCC I	*referent*	
	AJCC II	1.93	(1.67–2.24)
	AJCC III	3.48	(3.03–3.99)
	AJCC IV	14.44	(12.60–16.55)
	Unknown	4.94	(4.23–5.76)
**Morphology**	Adenocarcinoma (ADK)	*referent*	
	ADK in adenoma/polyp	0.73	(0.57–0.92)
	Mucinous ADK	1.06	(0.95–1.17)
	Carcinoids	0.42	(0.31–0.57)
	Carcinoma NOS	1.74	(1.53–1.99)
**Sublocation of Tumor**	Colon-Right	*referent*	
	Colon-Left	0.89	(0.82–0.97)
	Rectum	0.98	(0.91–1.07)
	Colon NOS	1.47	(1.31–1.65)

Abbreviations: ADK, Adenocarcinoma; AJCC, American Joint Committee on Cancer; CI, Confidence Interval; HR, Hazard Ratio; NOS, Not Otherwise Specified.

a. Multivariate Cox Proportional Hazards: Adjusted for all variables shown as well as age group and year of diagnosis.

b. American Indian/Alaskan Native suppressed due to small numbers.

In the sub-analysis, restricting analysis to Nevada’s CRC cases diagnosed in AJCC stages I-III and adjusting for age, sex, race/ethnicity, and year of diagnosis, patients who were identified as not receiving stage-appropriate treatment had 2.47 times the risk of death (95% CI: 2.21–2.76) within 5 years than those who received stage-appropriate treatment. Moreover, death from CRC at these stages after adjustment for stage-appropriate treatment was 14% and 28% significantly higher in Southern and Rural Nevada, (HR: 1.14; 95% CI: 1.01–1.29) and (HR: 1.28; 95% CI: 1.03–1.58), respectively, than in the Northwestern region. (Table 6).

**Table 6:** Sub-analysis: Risk of death^a^ from CRC, restricted to AJCC stages I-III with known TAG status, by geographic region and TAG. Nevada, 2003–2013.

	Model 1		Model 2	
	HR	95% CI	HR	95% CI
**Nevada Region**				
Northwestern Nevada	*Referent*		*referent*	
Southern Nevada	1.20	(1.06–1.36)	1.14	(1.01–1.29)
Rural Nevada	1.37	(1.11–1.69)	1.28	(1.03–1.58)
**Stage-Appropriate Treatment**				
Received			*referent*	
Did not receive	-	-	2.47	(2.21–2.76)

Abbreviations: AJCC, American Joint Committee on Cancer; CI, Confidence Interval; HR, Hazard ratio.

a. Multivariate Cox Proportional Hazards: Model 1 adjusted for sex, age group, race/ethnicity, and year of diagnosis. Model 2 adjusted for all Model 1 variables and stage-appropriate treatment.

## Discussion

Our study documented CRC survival disparities between the state of Nevada and the overall US; the proportion of patients diagnosed with CRC that survived at least five years was much lower in Nevada than the SEER-18 national survival. Based on a recent study of CRC survival in 38 US states (Nevada not included) [27], our data suggest that, despite slight methodological differences, survival from colorectal cancer in Nevada may be **the lowest in the entire US**. Such a disparity for one state within a single nation merits public health attention; regardless of the state of residence, all Americans deserve equal opportunity for optimum health outcomes in the face of a cancer diagnosis. Thus, the current study provides baseline information critical to clinicians, public health professionals, and all relevant stakeholders as they attempt to discern why Nevada’s outcomes are vastly divergent from its neighboring Western states and make plans for remediation.

The current study also documents for the first time that a striking within-state regional disparity largely explains Nevada’s poor survival profile. The low CRC survival observed in populous Southern Nevada is driving the state’s overall poor survival profile; taken alone, CRC survival in Northwestern Nevada is on par with national CRC survival. Unfortunately, a similar regional survival disparity has previously been documented in Southern Nevada for breast cancer [28] and lung cancer [29]. Nevada’s disparate results from its Western neighbors is likely related to shortages of medical training centers and physicians, especially in the densely populated Southern Nevada. Until the recent opening of the University of Nevada Las Vegas School of Medicine in Fall 2017, Southern Nevada was the largest metropolitan area in the US without a public medical school [30]. It also ranked 46^th^ in the nation for rates of residency and fellowship placements [31]. Despite a 10% increase in physician-per-capita rate from 2005–2015 [32], Nevada ranked 48^th^ - 49^th^ of all US states for rates of active patient care physicians, active general surgeons and active primary care physicians per capita in 2015 [31]. However, physician-per-capita rates in Northwestern Nevada were 33% higher than in Southern Nevada [32]. Additionally, while Southern Nevada comprises approximately 73% of Nevada’s total population [10], it had only 53% of its radiation oncologists, 65% of its gastroenterologists, and 66% of its primary care physicians in 2015, which may have limited access to screening and treatment [32]. Efforts to recruit, train and retain medical professionals are urgently needed for Southern Nevada.

Particularly troubling from a clinical perspective was our finding in the sub-analysis that a significant portion of Nevadans with colorectal cancer did not receive stage-appropriate treatment, especially in Southern and Rural Nevada. Previous US studies have linked racial/ethnic minorities, lack of insurance, and low socioeconomic status to lower likelihood of receiving recommended cancer care according to guidelines [33]. In Nevada, future research should clarify the reasons why some patients are not receiving guideline-recommended stage-specific care. Reasons are likely complex and multi-faceted, including access to care, practice differences between physicians, patient amenability to treatment based on age and comorbidities, and personal patient choices. If needed, modifications to colorectal cancer care protocols by clinicians should be implemented. Interventions should be developed to reduce barriers to receipt of quality care. Likewise, completeness and quality of cancer reporting, especially on treatment variables, should be improved to facilitate development of a more comprehensive picture of Nevada’s CRC survival profile.

One modifiable factor with the potential for leading to better CRC survival outcomes is colon cancer screening on a population basis, as it can detect tumors at earlier stages [34]. Screening can also prevent malignancies through the detection and removal of pre-cancerous polyps [34]. However, in Nevada, prevalence of CRC screening for 2002–2010, a reasonable approximation for our study period, was just 58%, ranking 45th out of 50 states, and well below the national CRC screening prevalence at that time of 65% [35]. Moreover, and likely contributing to the within-state survival disparities seen here, CRC screening uptake differs between the geographic regions of Nevada. Behavioral Risk Factor Surveillance System (BRFSS) estimates show that, similar to national trends, CRC screening in the Reno Metropolitan Statistical Area, (approximating Northwestern Nevada), increased from 49% in 2002 to 69% in 2012. However, the Las Vegas Metropolitan Statistical Area, (approximating Southern Nevada), only increased from 46% to 59% [36]. Additional resources should be committed to the development and implementation of CRC screening programs to increase uptake in Southern Nevada.

Among Nevada’s CRC cases, between 80–90% of each minority population (except American Indian) resided in Southern Nevada, a region shown to be disadvantaged. We found a lack of racial disparity between non-Hispanic blacks and Filipinos compared to non-Hispanic whites in Nevada for CRC survival, which runs counter to most other studies conducted in the US [37–40]. However, given that all racial/ethnic groups in Nevada had lower CRC survival than their national counterparts, this likely reflects the poor survival of the referent non-Hispanic white group rather than highlighting any notable progress in eliminating disparities for minorities. A similar absence of black-white survival disparities was previously documented for both cervical cancer [41] and lung cancer in Nevada [29]. Importantly, while other studies have consistently documented better CRC survival for the aggregated Asian/Pacific Islander group compared to non-Hispanic whites [3, 42], our disaggregation of Filipinos as distinct from Other APIs revealed that Filipinos do not have a survival advantage. Similarly, a previous study in California among distinct Asian groups found that Filipinos had significantly lower CRC survival than Chinese, Japanese, and non-Hispanic whites [43]. Thus, Filipinos, especially those in Southern Nevada, may benefit from targeted and culturally specific CRC prevention programs, including colon cancer screening.

Other factors impacting CRC survival in Nevada were consistent with the literature. Survival was slightly better in the more recent time period. With improvements in treatment modalities as well as screening detection of tumors at earlier stages, the number of CRC survivors in the US has increased, numbering almost 1.5 million in 2016 [1, 44]. Also consistent with previous research [45–48], a dose-response relationship was seen between patient SES status—previously linked to access to quality medical care, less aggressive treatment and delays in treatment [49, 50]—and CRC survival in the current study. We also saw better survival for women, especially those who were married. Several explanations for the survival advantage [51–53] of women for most cancers have been proposed, including sex-specific differences in risk factor prevalence, comorbidities and/or health seeking behaviors [52, 53] as well as sex hormones [54]. Married patients in Nevada, like the US [55–57], had greater survival than single, separated, or widowed; this phenomenon is often linked to greater social support, financial benefits and better health insurance [58, 59]. Lastly, one clinical factor that conferred better CRC survival was tumor location on the left side of the colon, also previously documented [60–62].

A major strength of this study is the large sample size provided by the Nevada Central Cancer Registry, enabling the creation of stable survival estimates by sex, race/ethnicity, stage of diagnosis, and region of Nevada for comparison purposes. Matching vital status with the NDI to completely capture deaths, customarily done in some other states, enhanced the accuracy of our survival estimates.

Nonetheless, this study is subject to a few limitations. Incompleteness of the data for key prognostic variables, such as stage at diagnosis, could have influenced the results of this study [63, 64]. In the sub-analysis, incompleteness of treatment variables precluded assessment of receipt of stage-appropriate treatment for many patients. Additionally, the quality of treatment data, especially receipt of chemotherapy and radiation, in cancer registries has not been thoroughly assessed across the United States [65, 66]. Individual level data was not available for SES; our measure by zip code is subject to some degree of ecological fallacy. Registry data does not include comorbidities, an important determinant of CRC survival; research has shown that patients with many comorbidities have worse outcomes [67, 68]. However, our use of cause-specific survival (rather than overall survival) partially compensates for the lack of comorbidity information. Additionally, we were unable to examine tumor markers and/or molecular subtypes that negatively influence survival [69]. Individual-level CRC screening data is not available from registries; we used BRFFS estimates of CRC screening by geographical area. Lastly, in US cancer registries, foreign-born populations have more missed deaths, especially in a state like Nevada that uses the “presumed alive” method for censoring [70]. Thus, the survival for groups comprised of many immigrants, including Filipinos, Hispanics, and Other APIs, may be overestimated.

In conclusion, this study accurately characterized for the first time the colorectal cancer survival profile in Nevada, revealing disproportionately poor survival compared to the US. We further identified demographic, social, and clinical factors associated with CRC survival. The identification of these determinants should help cancer prevention and control efforts, whether by seeking to intervene on modifiable determinants or providing programming to specific population groups. Certainly, CRC screening outreach efforts must continue and expand. A significant disparity between regions of Nevada was identified, disproportionately impacting rural and Southern Nevada, likely linked to access to care. Further studies are warranted in order to determine the barriers to receipt of stage-appropriate treatment for colorectal cancer patients in Nevada. Collaboratively, all stakeholders, including clinicians and researchers, must aggressively approach any opportunities not only for primary prevention of colorectal cancer, but also to maximize the survival potential, at least reaching national levels, for all patients who are diagnosed with colorectal cancer in Nevada. Certainly, Nevada should not have outcomes so vastly disparate from the rest of the American states.
